# Oral health investigations of indigenous participants in remote settings: a methods paper describing the dental component of wave III of an Australian Aboriginal birth cohort study

**DOI:** 10.1186/1472-6831-8-24

**Published:** 2008-08-15

**Authors:** Lisa M Jamieson, Susan M Sayers

**Affiliations:** 1 Australian Research Centre for Population Oral Health, The University of Adelaide, South Australia 5005, Australia; 2 Menzies School of Health Research, Institute of Advanced Studies, Charles Darwin University, Northern Territory 0811, Australia

## Abstract

**Background:**

A prospective Aboriginal Birth Cohort (ABC) study has been underway in Australia's Northern Territory since 1987. Inclusion of oral epidemiological information in a follow-up study required flexible and novel approaches with unconventional techniques. Documenting these procedures may be of value to researchers interested in including oral health components in remotely-located studies. The objectives are to compare and describe dental data collection methods in wave III of the ABC study with a more conventional oral health investigation.

**Methods:**

The Australian National Survey of Adult Oral Health (NSAOH) was considered the 'conventional' study. Differences between this investigation and the dental component of the ABC study were assessed in terms of ethics, location, recruitment, consent, privacy, equipment, examination, clinical data collection and replication. In the ABC study, recording of clinical data by different voice recording techniques were described and assessed for ease-of-use portability, reliability, time-efficiency and cost-effectiveness.

**Results:**

Conventional investigation recruitment was by post and telephone. Participants self presented. Examinations took place in dental clinics, using customised dental chairs with standard dental lights attached. For all examinations, a dental assistant recorded dental data directly onto a laptop computer. By contrast, follow-up of ABC study participants involved a multi-phase protocol with reliance on locally-employed Indigenous advocates bringing participants to the examination point. Dental examinations occurred in settings ranging from health centre clinic rooms to improvised spaces outdoors. The dental chair was a lightweight, portable reclining camp chair and the dental light a fire-fighter's head torch with rechargeable batteries. The digital voice recorder was considered the most suitable instrument for clinical dental data collection in the ABC study in comparison with computer-based voice-recording software.

**Conclusion:**

Oral health examinations among indigenous populations residing in predominantly remote locations are more logistically challenging than are surveys of the general population. However, lack of resources or conventional clinical infrastructures need not compromise the collection of dental data in such studies. Instead, there is a need to be flexible and creative in establishing culturally-sensitive environments with available resources, and to consider non-conventional approaches to data gathering.

## Background

Aboriginal people in Australia have the poorest health and highest premature mortality rates of any indigenous minority in the first world [1]. More than 25 percent of Aboriginal adults are obese [2] and 10–30 percent have Type II diabetes with early age of onset [3]. End stage renal disease rates are the highest reported in the world [4] and indigenous Australians are 1.3 times more likely than non-indigenous people to report heart disease and/or circulatory problems [2]. As a result of these conditions, the life expectancies of 59 years for Aboriginal males and 65 years for Aboriginal females [5] are approximately 17 years less than for non-Aboriginal people in Australia and similar to the United Nations Children's Fund 2007 estimate of 65 years for developing countries [6].

Oral health is recognised as playing an important role in general health and well-being [7]. Oral diseases are not only major causes of infection and tooth loss, but cause debilitating pain and difficulties with eating/speaking, as well as limit social interactions [8]. The impact of oral disease is not confined to the mouth, with associations between chronic oral infections and cardiovascular diseases [9], diabetes [10], stroke [11] and pre-term low birth weight [12] being established. Indigenous Australians have markedly higher levels of dental disease than their non-indigenous counterparts [13], with the prevalence of tooth loss due to periodontal diseases being particularly high [14].

In 2004–2006, a National Survey of Adult Oral Health (NSAOH) was undertaken among a representative sample of adult Australians to determine the prevalence and extent of dental diseases and related oral conditions at a national level [15]. The survey involved two quantitative methodologies: a self-report socio-dental questionnaire (subjective tool) and dental epidemiological examinations (objective tool). Although the survey aimed to be representative, only 1.4 percent of participants identified as being indigenous [15] compared to 2.0 percent in the estimated resident population [16]. This suggests that the conventional methodologies utilised in the national survey were perhaps inappropriate for indigenous populations, a point noted in other national health surveys with poor indigenous representation [17].

An Aboriginal Birth Cohort (ABC) has been established in the Northern Territory of Australia since 1987 based on the principle that health maintenance and disease development is a dynamic process that occurs over the total life span [18]. As part of this life course study, an assessment of oral health was included in the follow-up conducted at a mean age of 18 years. Although this cohort was recruited at a single hospital point at birth, at follow-up most participants lived in vast, sparsely populated areas with poor infrastructures. Almost 80 percent of assessments were conducted in remote settings with no dental assistance available. Unconventional methodologies to ensure appropriate recruitment and data collection for the dental component were thus necessary due to the multiple logistic and cultural challenges.

Documenting the data collection techniques used in the dental component of the ABC study follow-up may benefit researchers collecting oral health data in remote and/or indigenous health studies. The objectives of this paper are: (i) to describe dental data collection methods in wave III of the ABC study compared with the conventional oral health investigation of NSAOH and; (ii) to assess different methods of recording dental epidemiological information in the ABC study using digital and computer-based voice recording techniques.

## Methods

The ABC study is a life course investigation that, in its third phase, involved participants aged 16–20 years residing in more than 40 different communities throughout the Northern Territory's Top End. Data collected in wave III included socio-demographic, anthropometric, renal, metabolic, cardiovascular, haematological, infection, social and emotional well-being and dental items.

The dental component of the ABC study was based on the same oral epidemiological and social survey methodology as NSAOH [15], which was conducted concurrently. For purposes of this paper, NSAOH was considered to be a 'conventional' dental epidemiological investigation while the dental component of the ABC study was considered to be 'non-conventional'. Differences between the dental component of the ABC study and the conventional study were assessed in terms of ethics, location, recruitment, consent, privacy, equipment, examination, clinical data collection and replication.

### Data recording in the ABC study

Because it was not possible to have a dental assistant record the clinical dental data in the ABC study, three different data recording techniques were investigated. These included a small, hand-held digital voice recorder that operated via battery and allowed instant replay for data to be transcribed; computer-based voice-recording software using Microsoft Word^© ^facilities, which required a lap-top computer and training for the software to recognise and transcribe the operator's voice into a Microsoft Word^© ^document; and computer-based voice-recording software using Microsoft Excel^© ^spreadsheets, which again required a lap-top computer and voice-recognition training, but in addition the use of prompts to guide between different cells in the Microsoft Excel^© ^template. The three dental data collection methods were assessed against a human voice recorder gold standard using a Likert-type scale to describe general ease-of-use, technical ease-of-use, portability, reliability, time-efficiency and cost-effectiveness. Assessments were conducted both in an office setting and in the field.

## Results

### Ethics

Ethical approval for the conventional study was received by the University of Adelaide's Human Research Ethics Committee. Ethics approval for wave III of the ABC study was received by the Ethics Committee of the Northern Territory Department of Health and Community Services and Menzies School of Health Research, which includes an Aboriginal sub-committee with absolute right of veto.

### Location, privacy and equipment

Dental examinations in the conventional investigation took place in dental clinics, using customised dental chairs with standard dental lights attached (Table 1). Privacy was afforded through dental surgery walls. The examinations were conducted using non-disposable probes and a mirror light kit with disposable mirror heads and disposable plastic sleeves. By comparison, dental examinations in the ABC (non-conventional) study occurred in a range of opportunistic settings including hospital rooms, health centres, community recreation halls, community sheds, school classrooms, women's centres and outdoor spaces. Privacy was ensured through creative use of twine and curtains, and any additional props that may have been present (for example, up-ended tables, racks of second-hand clothing). The dental chair was a portable reclining camp chair (total weight 5 kg) and the dental light a fire-fighter's head torch with rechargeable batteries. The probes used for examination were disposable, as were the mirror heads and plastic sleeves used with the mirror kit (the same as those used in NSAOH). All items were portable and disposable, with a total weight of 8 kg. Logistic constraints to routine sterilisation procedures in the field made it difficult to use non-disposable probes in the ABC study.

**Table 1:** Comparison of conventional (NSAOH) with non-conventional (ABC study) dental data collection techniques

	Conventional	Non-Conventional
Study	National Survey of Adult Oral Health (NSAOH)	The dental component of the Aboriginal Birth Cohort (ABC) study
Design	Cross-sectional, utilising a three-stage, stratified clustered sampling design	Prospective longitudinal
Measures	Predominantly dental	Multidisciplinary (anthropometric, respiratory, renal, metabolic, cardiovascular, haematological, infection, social and emotional well-being, dental)
Location	All Australian states and territories	40 communities in Northern Territory's top end; nearly 80% of participants remotely-located
Recruitment	Postal contact, telephone	Community-employed 'locators', telephone, house visits, relative visits, school visits
Number of contacts made	Up to 6 for telephone interview, up to 2 for clinical examination	Endless, or until the team left the community or an outright refusal was given
Transport	Participants' responsibility	Participants would be picked up and dropped off
Consent	Individual verbal and written consent	Community permit, individual written consent
Participant age range	15 years+	16–20 years
Setting	Public dental clinics in participants' post codes	Range including hospital rooms, community health clinics, women's centres, recreation halls, school classrooms, outdoors
Privacy	Dental surgery walls	Sheets, tables, racks of clothing, table cloths
Dental chair	Dental surgical chair	Portable, reclining camp chair
Light	Dental light attached to chair	Fire-fighter's head torch with rechargeable batteries
Instruments:		
Examination probe	Non-disposable periodontal probe with 2 mm markings	Disposable periodontal probe with 2 mm markings
Mirror	Mirror light kit with disposable mirror heads and disposable plastic sleeve	Mirror light kit with disposable mirror heads and disposable plastic sleeve
Data recording	Dental assistant entering data directly onto laptop computer	(i) Digital voice-recorder (ii) Computer-based voice-recording software using Microsoft Word^© ^facilities and; (iii) Computer-based voice-recording software using Microsoft Excel^© ^spreadsheets.
Replication	Conducted with 157 participants to assess reliability of 29 examiners	Not possible

### Recruitment, number of contacts made and transport

A three-staged, stratified clustered sampling design was implemented in NSAOH to select Australian residents aged 15+ years. The sampling frames were households with listed telephone numbers recorded in an electronic white pages database. The first stage selected postcodes, the second stage selected households within sampled postcodes, and the third stage selected one person aged 15+ years from each sampled household. Approximately 10 days before dialling each sampled telephone number, a primary approach letter explaining NSAOH's purpose was mailed to the address accompanying the sampled telephone numbers. Up to six contact calls were made. At the completion of each telephone interview, participants were requested to take part in a clinical oral epidemiological examination, with appointments made at public dental clinics within or near the postcode in which people were sampled. Participants were expected to make their own way to the clinic. A second appointment was made if participants failed to present at the first appointment.

In wave III of the ABC study, a letter of introduction was sent to each Aboriginal community council explaining the purpose of the study, requesting assistance in locating a suitable space in which to work, and enquiring as to the availability of a local person willing to be employed in one or all roles of locator, translator and advocate. A permit to visit the community was applied for and lists of potential participants were shown to community councils and/or health centres. In a given community, strategies employed to locate participants; primarily through the locator, included canvassing high schools, homes, work places and recreation areas. Participants would usually be collected and dropped off by study members in a vehicle, and were encouraged to bring along any children or supportive family members. In the urban settings, participants were canvassed in a similar manner, although there was a greater reliance on telephone calls and house calls. Snowball techniques were also employed. A considerable number of attempts were made to contact participants, only ceasing when the study team left the community or were given an outright refusal from a study member.

### Consent

Participants in NSAOH provided verbal consent prior to answering questions in the telephone interview and signed informed consent prior to the oral examination. Signed, informed consent was received for the individual components of the ABC study, including permission to take a photograph for future identification purposes. The male Aboriginal research assistant involved in the study went through a visual enhanced staged consent for male participants, while female study team members and/or female locally employed assistants went through the consent process with female participants.

### Examinations

The dental examinations in both the NSAOH and ABC study recorded presence or absence of oral mucosal lesions; tooth loss; gingivitis, calculus and plaque levels; periodontal status; caries experience; attrition and dental fluorosis (Table 2). The collection of buccal mucosal cells (for DNA purposes) and gingival crevicular fluid was implemented only in NSAOH, while dental trauma was assessed only in the ABC study.

**Table 2:** Oral epidemiological measures used in NSAOH and the ABC study

Measure	Description	Included in NSAOH	Included in ABC study
Oral mucosal lesions	Presence of selected mucosal lesions	√	√
Tooth retention	Presence/absence of all teeth including presumptive cause of tooth loss	√	√
Gingivitis and calculus	Bleeding on probing and calculus detected at 6 index sites	√	√
Plaque	Presence/absence of visible plaque at 6 index sites	√	√
Periodontal destruction	Probing pocket depth and gingival recession recorded at two sites of all teeth.	√	√
Caries experience	Presence of decay or restoration recorded for all coronal surfaces	√	√
Attrition	Incisal tooth wear measured for anterior teeth	√	√
Dental trauma	Presence of past or present trauma to the enamel or dentine of anterior teeth	x	√
Fluorosis	Presence of fluorosis in the upper central incisors	√	√
Buccal mucosal cells	DNA testing	√	x
Gingival crevicular fluid	Presence/absence of inflammatory markers	√	x

### Data recording

A dental assistant was present for all NSAOH examinations to record dental data directly into a screen-based entry system on a laptop computer, in comparison with the ABC study where three dental data recording methods were trialled. When compared with the gold standard of a human voice recorder, the digital recorder scored highly for portability and cost-effectiveness and moderately for general and technical ease-of-use, reliability and time-efficiency (Table 3). The computer-based voice recorder with Microsoft Word^© ^facilities scored poorly in general and technical ease-of-use and portability, and very poorly in reliability and time-efficiency. The computer-based voice-recording software using Microsoft Excel^© ^was worst, with low points scored in general and technical ease-of-use, reliability and time-efficiency. Of note the human voice recording method scored poorly for portability and cost-effectiveness, for example, an extra seat on charter planes being necessary thus substantially increasing the cost. The main issue with the digital recorder was time efficiency, with time required to play back the recording and to transcribe the information onto the paper-based data capture forms. However, this was even more so for the computer-based voice recording software, where the operator needed to consistently check the screen for wording, to repeat phrases and to, in turn, transcribe the data onto the paper-based forms. Both computer-based systems proved unreliable under time pressure, particularly when electricity was unavailable or there was not enough computer memory.

**Table 3:** Comparison of dental data recording techniques in wave III of the ABC Study

	Human recorder (gold standard)	Digital voice recorder	Computer-based voice-recording software using Microsoft Word^© ^facilities	Computer-based voice-recording software using Microsoft Excel^© ^spreadsheets
General ease-of-use	••••	•••	•	•
Technical ease-of-use	••••	•••	••	•
Portability	•	••••	••	••
Reliability	•••	•••	•	•
Time-efficiency	••••	•••	•	•
Cost-effectiveness	•	••••	•••	•••

Key: •••• excellent; ••• good; •• acceptable; • poor

### Replication

In NSAOH, replicate examinations were conducted with 157 participants to assess reliability of 29 examiners and moderate agreement was reached for measures of decayed dental surfaces (median intra-class correlation = 0.56). As the dental component was only part of an extensive assessment taking over two hours, research fatigue considerations, time and logistical constraints, made it not possible to conduct replicate dental examinations in the ABC study. In the Dunedin Multidisciplinary Health and Development Study, it has also been reported that replicate oral examinations are not possible because of time constraints [19], indicating that this is perhaps a common issue in longitudinal life course studies in which oral health data is not the only information gathered and co-operation with future follow-up is being fostered.

### Data cleaning

There were a number of data cleaning steps undertaken in the ABC study prior to entry into statistical software packages. These included cleaning when data was in its hardcopy form (on-site by a research assistant at the end of each data collection day) and once data had been entered into an electronic spreadsheet. Later double entry of 100 dental data forms was undertaken with a 5% error rate confined to one variable that was corrected in the final database subsequently used for all statistical analysis.

## Discussion

### Logistical aspects

The collection of dental information in health studies based in remote settings need not be compromised because of lack of access to conventional resources or examination facilities. It is possible to obtain light and portable dental chairs, head torches and disposable instruments to ensure high quality examinations take place. It is also possible to have cost-effective and portable recording equipment to enable reliable collection of clinical data.

In any given multidisciplinary health investigation such as the ABC study, it is critical that the materials, instruments and data collection tools used in the dental component are minimal, light, fool-proof, durable, expendable and able to be set down and packed up again quickly in a range of environments whilst still allowing the privacy of participants to be respected. A key issue for the ABC study was portability of study equipment, with travel including ferry, four-wheel-drive and charter plane. There was limited space and sometimes weight constraints on all transport modes, and equipment from other components of the study were bulky (for example, centrifuge and dry shipper for blood samples). Health investigations that involve a remote component such as the ABC study may face additional logistic challenges in terms of weather (heavy rain, floods, cyclones), transport (unavailable or inoperable charter planes or vehicles) or unforeseen road closures; it is important that the equipment used, including dental equipment, does not add any additional duress to these logistical elements.

Disposable probes were used for examination because routine sterilisation procedures were unable to be undertaken in the field due to the multiple and variable examination sites in the follow-up with poor supporting infrastructures as well as the cost involved and time available. Disposable probes have been used in other oral epidemiological studies and are sanctioned by the Australian Research Centre for Population Oral Health for use in settings that are not conducive to routine sterilisation procedures.

At a community level, logistical challenges in the ABC study usually involved locating a convenient location in which to conduct assessments, and organising and picking up a reliable vehicle to collect and drop off study participants. The conventional study, in comparison, faced markedly fewer logistical constraints. Postal techniques and telephone calls were largely successful, participants did not need to be picked up and dropped off, and examinations were conducted in dental clinics where infrastructure (such as electricity and privacy) was generally reliable.

### Cultural aspects

There were a number of cultural aspects in the ABC study that needed to be acknowledged and addressed in order for recruitment and dental data collection to be successful. Participants were highly mobile, meaning marked effort was necessary in order for a) participants to be located and b) participants agreeing to present for data collection. While this is a feature of health research conducted among indigenous populations on the whole [20], it also reflects the transient nature of the early adult age-group being assessed. There was poor voluntary presentation, perhaps due to the unfamiliar and somewhat invasive nature of the data collection procedures (with many participants having never visited a dentist before) and possibly because the study team were predominantly non-indigenous [21]. The study team needed to be sensitive to community circumstances such as deaths and funerals, as well as unforeseen events such government interventions [22]. Using local advocates was essential to engender a spirit of trust in the community, to help build a rapport with participants and to enable study team members to communicate with those for whom English was a second language. Consent documents were not provided in any language aside from English, due to the large number of language groups used by study participants. If it appeared that English was not the first language of the study participant, and that the participant was struggling to understand the consent process, the locally-employed research assistants, who did speak the same first language, were asked to convey the consent information verbally. Other study participants with a solid grasp of English also assisted.

Gender separation was critical at times when female participants were present with children and when various skin-groups could not be present in the same vicinity [23]. Timing in the day was also crucial; early morning was non-ideal for late-rising communities, and mid-afternoon no use in communities where card playing was a strong social feature [24]. Because many participants were not familiar with the dental instruments, time needed to be taken to explain what was going to be done and how instruments would be used. When children were present, it helped to let them wear the dental headlamp for a time, to show them the dental mirror and to examine their own teeth if time permitted.

Many participants required assistance with the self-report component of the dental examination, with the questionnaire structure being unfamiliar to some (the use of Likert-type scales, for example) and many not wanting to lose face by showing that they did not fully understand the questions (literacy levels of indigenous Australians being markedly less than their non-indigenous counterparts [25]). For many participants, especially those remotely-located and for whom English was a second language, the concepts used in the questionnaire were frequently not something they were familiar with (items relating to dental fear, self-rated oral health for example). Because the dental component was one of nine components being measured (and was often the last), participants were often exhausted by the time they reached the dental station and were impatient to leave (especially if accompanied by young infants). A large number of research investigations being conducted in some communities also meant that participants were 'surveyed out', particularly if they felt that nothing was being returned to either them or the community as part of participating in such studies [26]. Each participant received food and a 'thank you' bag containing, among other items, a toothbrush, toothpaste sample and pamphlet outlining the risk of smoking in periodontal disease and oral cancer. Towards the end of the follow-up wave urban participants received a local department store voucher to boost recruitment, however we were unable to show if this made a difference to presentation. Each community received a de-identified summary report of the community-level findings.

The cultural aspects of the conventional study, in comparison with the ABC study, were relatively less, although issues regarding English as a second language (13 percent of the sample spoke another language other than English at home [15]) and exhaustion at the end of the dental examination did exist.

### Data collection

While a human recorder may be the best way of capturing dental epidemiological data in conventional settings, this may not be possible in investigations such as the ABC study where logistical or financial constraints prevent the inclusion of another person (no room on the charter plane etc). For purposes of this study, digital voice recorders proved to be portable, cheap, easy-to-use, reliable and did not require much time to implement. Computer-based voice recording software were comparatively time-consuming, unreliable, logistically difficult and burdensome, although it may be that such software becomes less cumbersome and more dependable in the future.

Computer-based voice-recording software has proved successful in clinical settings such as endoscopy [27], but other areas of the medical profession adopting this approach for data recording reported similar problems as those experienced in the ABC study. For example, in a radiology setting, Gale *et al*. [28] found that the time required to produce a report using voice-recording software was significantly longer than that required by the traditional tape-transcription system, with the additional cost in time estimated to be, for their institution, $100,000 annually. In the dental literature, computerised voice recognition methods have been found to be significantly slower than traditional examiner/recorder methods, although differences in error rates have not been substantial [29]. While it is likely that future systems will require less effort, in an investigation such as the ABC study, time limits for each dental examination need to be strictly adhered to. In this study, a digital voice recorder, which gave the examiner the option of playing back the recording at his or her leisure, was the preferred option when space limitations or other logistical constraints meant that inclusion of a human recorder was not possible.

## Conclusion

Oral health investigations among indigenous populations residing in predominantly remote locations are clearly more challenging than are surveys of the general population. A substantial number of cultural, ethical and logistical issues must be addressed. However, by using a range of creative and flexible data collection techniques, high-quality dental information may be collected in settings that differ from the conventional. In wave III of the ABC study, it was not practical to employ human voice recorders for the collection of dental data because of the extra space requirements, or computer-based voice-recording software because of the additional time required to record data and the unreliability of data capture. Digital voice recorders were the preferred choice, being portable, reliable, cheap, easy-to-use and requiring minimal time to implement. Despite the difficulties, it is imperative that investigations such as the ABC study continue to include oral health components, as it is only through such surveys that the prevalence and severity of oral conditions of under-represented populations such as indigenous Australians, particularly those in remote locations, can be assessed in order to inform appropriate policy and service provision.

## Competing interests

The authors declare that they have no competing interests.

## Authors' contributions

LMJ conducted the analysis and drafted the manuscript. SMS was responsible for the original design of the ABC study and participated in the writing and completion of the manuscript. Both authors read and approved the final manuscript.

## Pre-publication history

The pre-publication history for this paper can be accessed here:


